# Gynæcology

**Published:** 1879-04

**Authors:** 


### GYNÆCOLOGY.

INDICATIONS AND CONTRA-INDICATIONS FOR OVARIOTOMY IN THE TREATMENT OF OVARIAN CYSTS.—S. Duplay. (*Archives Générales de Médecine*, Jan., 1879.)

The following is the author's résumé of an interesting article on this subject:

1st. Before thinking of proposing the question of the indications and contra-indications of ovariotomy, the surgeon should have established as exact a diagnosis as possible, and have made an exploratory puncture.

2d. In relation to the time when ovariotomy should be proposed, I formally reject the early operation, and I believe that ovariotomy is only indicated when the cyst has become, by its volume, a cause of excessive inconvenience to the patient, or when it occasions, by local or general accidents, imminent danger to life.

3d. Late ovariotomy, although it should not be adopted, as a general rule, is not contra-indicated by the existence of the most grave local and general complications, such as peritonitis, inflammation, suppuration, gangrene of the cyst.

4th. Ovariotomy is absolutely contra-indicated in cases of cysts of the ovaries complicated by diseases, general or local, independent of the presence of the cyst, and capable of causing by their ulterior evolution the death of the patient.

5th. The divers local conditions dependent upon the state of the cyst (walls and contents), of its connections (adhesions), of the state of the peritoneum (ascites), are of but little importance as regards the indications and contra-indications of ovariotomy. I should, however, make two exceptions: first, relative to unilocular cysts, with serous contents, non-albuminous (cysts of the parovarium), in which ovariotomy, I think, as a general thing, is not indicated; second, relative to extensive adhesions to the uterus, bladder, rectum, etc., which, especially when accompanied by abundant ascites, often indicate a malignant affection. In these cases, without daring to prescribe ovariotomy absolutely, I would advise its postponement as long as possible.

6th. Finally, ovariotomy is applicable to cysts of the ovary complicated by pregnancy, when the life of the mother and child is directly menaced by the development of the tumor, and when tapping is without effect.

